# The impact of exercise on growth factors (VEGF and FGF2): results from a 12-month randomized intervention trial

**DOI:** 10.1186/s11556-019-0215-4

**Published:** 2019-06-24

**Authors:** Darren R. Brenner, Yibing Ruan, Scott C. Adams, Kerry S. Courneya, Christine M. Friedenreich

**Affiliations:** 1 Department of Oncology, Cumming School of Medicine, University of Calgary, Calgary, Alberta Canada; 2 Department of Community Health Sciences, Cumming School of Medicine, University of Calgary, Calgary, Alberta Canada; 3 Department of Cancer Epidemiology and Prevention Research, CancerControl Alberta, Alberta Health Services, Holy Cross Centre – Room 513C, Box ACB, 2210 – 2nd St. SW, Calgary, Alberta T2S 3C3 Canada; 4 Faculty of Physical Education and Recreation, University of Alberta, Edmonton, Alberta Canada

**Keywords:** Aerobic exercise, Growth factors, Breast Cancer, Intervention trial, Randomized controlled trial

## Abstract

**Background:**

Vascular endothelial growth factor (VEGF) and Fibroblast growth factor-2 (FGF2) are angiogenic cytokines in normal tissues and tumors. Evidence suggests that increased growth factor expression in adipose tissue leads to improved vascularity and decreased hypoxia, fibrosis, and inflammation, which may, in turn, reduce post-menopausal breast cancer risk.

**Objective:**

We investigated whether or not exercise had dose-response effects on levels of plasma VEGF and FGF2 in postmenopausal women.

**Methods:**

Four hundred previously inactive but healthy postmenopausal women aged 50–74 years of age were randomized to 150 or 300 min per week of aerobic exercise in a year-long exercise intervention. VEGF and FGF2 were measured from fasting serum samples with a custom-plex multiplex assay.

**Results:**

A high compared to moderate volume of aerobic exercise did not cause chronic changes in plasma VEGF or FGF2 levels in intention-to-treat or per-protocol analyses.

**Conclusions:**

We did not detect differences in growth factor levels related to increasing doses of exercise. It is unlikely that changes in VEGF and FGF2 levels mediate the reduction in risk of post-menopausal breast cancer development in associated with increased levels of exercise.

**Trial registration:**

Clinicaltrials.gov identifier: NCT01435005.

## Introduction

Regular lifetime exercise has been consistently associated with a reduced risk of developing postmenopausal breast cancer [1]. Several mechanisms underlying the impact of exercise have been identified, including reductions in adiposity, sex hormone levels, insulin resistance and chronic inflammation [1]. These pathways explain some portion of the association; however, additional mechanisms require identification and further investigation.

One additional mechanism of interest is the impact of exercise on growth factors known to drive cell proliferation and tissue growth [2]. Vascular endothelial growth factor (VEGF) is a potent angiogenic cytokine in normal tissues and tumors. It has been shown to stimulate endothelial cell proliferation in vitro and induce angiogenesis in vivo [3]. Fibroblast growth factors are a family of growth factors related to a broad range of physiologic functions including angiogenesis and epithelial proliferation [4]. Fibroblast growth factor-2 (FGF2) is an interesting candidate in breast cancer pathology as a common single nucleotide polymorphism in the gene has been associated with breast cancer risk in large studies among Western populations [5].

In a previous investigation from the Breast cancer and Exercise Trial in Alberta (BETA), we observed that higher levels of activity were associated with reduced body fat [6]. Unhealthy fat pad expansion is characterized by inadequate vascularization and hypoxia – leading to increased fibrosis, inflammation and insulin resistance [7]. We hypothesized that exercise-induced change in adipose tissue vascular remodeling may account for some of the observed exercise-related reduction in postmenopausal breast cancer risk. As part of ancillary investigations from BETA to evaluate novel mechanisms relating exercise to breast cancer risk, we investigated whether a higher versus a more moderate volume of exercise would lead to differential changes in VEGF and FGF2 levels.

## Materials and methods

BETA study design and eligibility criteria have been described elsewhere [8]. Briefly, we randomized 400 postmenopausal women, aged 50–74 years old, who were previously inactive but otherwise healthy to 150 (MODERATE) or 300 (HIGH) minutes/week of aerobic exercise at 65–75% of maximal heart rate reserve for 12 months. Participants completed three supervised sessions and two unsupervised sessions per week, with the HIGH volume exercise group completely exercise sessions that were twice as long as the MODERATE volume group. During exercise sessions, any aerobic activity that raised that participants’ heart rates to their target heart rate zones was permitted.

Fasting blood samples were drawn from study participants at baseline, 6 and 12 months, after a 24-h complete abstinence from exercise and alcohol intake and at least 10 h since the last food intake. FGF2 and VEGF levels in plasma were quantified using a custom-plex Human Circulating Cancer Biomarker Multiplex Assay. The multiplex assays were performed at Eve Technologies (Calgary, AB, Canada) using the Bio-Plex™ 200 system (Bio-Rad Laboratories, Inc., Hercules, CA, USA). The detection limits for FGF2 and VEGF in this assay were 3.6 and 6.4 pg/mL, respectively. The inter- and intra-assay coefficients of variation (CV) were 6.3 and 7.9% for FGF2, and 10.2 and 12% for VEGF, which are below or comparable to other CVs using the Bio-Plex platform [9]. All lab personnel were blinded to the intervention assignment. Each participant’s samples at different time points were analyzed in the same batch and each batch included an equal number of MODERATE and HIGH blood samples.

Changes in concentrations of growth factors in the two intervention groups were compared in an intention-to-treat (ITT) analysis using linear mixed models that accounted for repeated measures of FGF2 and VEGF at 6 and 12 months, adjusting for baseline levels of the biomarker as a covariate. Treatment effect ratios (TERs) were calculated from these models and denoted the HIGH: MODERATE ratio of geometric mean biomarker levels over 12 months (reflecting changes from baseline to 6 and 12 months). The ITT analysis was repeated by stratifying on a priori potential effect modifiers: baseline body mass index (BMI), baseline fitness (estimated VO_2max_) and total percent body fat (full body dual X-ray absorptiometry scan).

A per-protocol analysis was undertaken for women who completed, on average, 90 to 100% of the exercise prescription for the MODERATE group or ≥ 90% of prescription for the HIGH group from weeks 13–52.

## Results

A total of 386 women completed the trial with no statistically significant difference in dropout between arms. Baseline characteristics and exercise adherence of the BETA study participants have been previously described [6]. We observed no statistically significant effects for increasing quartiles of exercise and change in circulating levels of VEGF or FGF2 with both groups combined (non-randomized, results not shown).

Results from linear mixed models suggested overall lower, but non-statistically significant group differences between baseline and 12 months for both FGF2 and VEGF for both arms of the trial. The HIGH group experienced slightly larger changes than the MODERATE group, but these changes were not clinically relevant (FGF2: TER 0.98, 95% CI 0.94–1.02; VEGF: TER 0.94, 95% CI 0.86–1.02; Table 1). Furthermore, the per-protocol analysis found essentially no differences between baseline and end of intervention effects with the TERs approximately equal to 1.0 for both biomarkers. Similarly, a per-protocol analysis stratified by exercise intensity, i.e., whether participants achieved 60% of the prescribed time in the target heart rate zone, showed no significant group differences in FGF2 and VEGF (Table 2).

**Table 1:** Intention-to-treat and per-protocol analysis of FGF2 and VEGF concentrations between high and moderate volume exercise groups in BETA (*n* = 386)

Group	n	Baseline		6 Months		12 Months		Treatment Effect		Between-Group P ^c^
		Geo-metric Mean ^a^	95% CI	Geo-metric Mean	95% CI	Geo-metric Mean	95% CI	Ratio of High/Moderate ^b^	95% CI	
Intention-to-treat										
FGF2										
High	195	121.3	114.4–128.5	116.1	110.1–122.4	123.3	117.7–129.2	0.98	0.94–1.02	0.28
Moderate	191	118.8	111.9–126.2	122.9	116.2–130.0	120.1	113.1–127.6			
VEGF										
High	195	62.3	55.1–70.4	58.5	51.7–66.3	58.6	51.3–66.9	0.94	0.86–1.02	0.15
Moderate	191	64.9	57.2–73.6	63.6	55.7–72.6	67.3	58.9–76.9			
Per-protocol ^d^										
FGF2										0.64
High	80	119.8	110.8–129.4	116.0	107.3–125.3	121.2	113.5–129.4	1.02	0.95–1.10	
Moderate	58	112.6	100.0–126.7	113.9	101.9–127.3	109.6	98.3–122.2			
VEGF										0.74
High	80	60.6	50.8–72.3	60.6	50.0–73.4	58.0	47.6–70.7	0.98	0.84–1.13	
Moderate	58	70.3	54.2–91.0	64.9	49.9–84.6	74.0	56.9–96.3			

a. Geometric mean of FGF2 and VEGF was in the unit of pg/mL

b. The geometric mean ratios were estimated from least square means for the difference in treatment effect between high and moderate volume exercisers averaged across the entire study period adjusted for the baseline values and then back log-transformed

c. *P* value corresponds to the null hypothesis that the ratio of treatment effect between high- and moderate-volume groups equals 1 against the 2-sided alternative hypothesis

d. Women assigned to the moderate-volume group were adherent if they completed 90 to 100% of the exercise prescription (mean, 135–150 min/wk), weeks 13 to 52 at full prescription; women assigned to the high-volume group were adherent if they completed at least 90% of the exercise prescription (mean, ≥270 min/wk), weeks 13 to 52 at full prescription

**Table 2:** Stratified analyses of changes in FGF2 and VEGF between high and moderate volume exercise groups in BETA (*n* = 386)

Stratification variables	Prescribed exercise duration	n	6 months percent change from baseline	12 months percent change from baseline	Treatment effect ^a^		Between-group P ^b^
					Ratio of HIGH:MOD	95% CI	
FGF2							
Time at high intensity ^c^							
< 60% prescribed	MODERATE	17	2.0	4.9	1.03	0.91–1.17	0.62
	HIGH	44	0.4	0.3			
≥ 60% prescribed	MODERATE	41	0.9	−5.6	1.01	0.92–1.12	0.78
	HIGH	36	−7.4	2.3			
BMI ^d^							
< 30	MODERATE	116	9.0	6.8	0.99	0.94, 1.05	0.83
	HIGH	121	4.0	5.2			
≥ 30	MODERATE	75	5.1	2.2	0.95	0.88, 1.02	0.13
	HIGH	74	−7.3	6.3			
VO_2max_ level ^e^							
< 27.2 ml/kg min	MODERATE	93	8.2	1.3	0.94	0.88, 1.00	0.05
	HIGH	98	−2.2	8.2			
≥ 27.2 ml/kg min	MODERATE	98	6.7	8.5	1.02	0.96, 1.08	0.56
	HIGH	97	1.2	3.1			
Total body fat ^f^							
< 29.7 kg	MODERATE	94	9.2	5.5	1.02	0.96, 1.08	0.53
	HIGH	99	1.2	3.0			
≥ 29.7 kg	MODERATE	97	5.8	4.5	0.94	0.88, 1.00	0.04
	HIGH	96	−2.2	8.5			
VEGF							
Time at high intensity							
< 60% prescribed	MODERATE	17	−6.5	11.0	1.04	0.83–1.31	0.72
	HIGH	44	5.0	7.2			
≥ 60% prescribed	MODERATE	41	−8.0	3.2	0.90	0.73–1.10	0.28
	HIGH	36	−6.0	−16.8			
BMI							
< 30	MODERATE	116	−4.1	6.0	0.90	0.80, 1.01	0.06
	HIGH	121	−10.4	−10.6			
≥ 30	MODERATE	75	1.5	0.3	1.01	0.88, 1.15	0.90
	HIGH	74	1.4	2.9			
VO_2max_ level							
< 27.2 ml/kg min	MODERATE	93	4.0	1.9	0.93	0.82, 1.06	0.29
	HIGH	98	−1.8	−6.7			
≥ 27.2 ml/kg min	MODERATE	98	−7.2	5.7	0.94	0.83, 1.06	0.30
	HIGH	97	−10.2	−5.1			
Total body fat							
< 29.7 kg	MODERATE	94	−7.7	4.5	0.92	0.80, 1.06	0.25
	HIGH	99	−9.1	−10.5			
≥ 29.7 kg	MODERATE	97	4.0	3.1	0.95	0.85, 1.06	0.39
	HIGH	96	−3.0	−0.3			

a. HIGH:MODERATE ratio of geometric means for biomarker levels over 12 months adjusted for biomarker level at baseline. A ratio < 1.0 indicates lower biomarker concentrations in the HIGH exercise group at 6 and 12 months; a ratio > 1.0 indicates lower biomarker concentrations in the MODERATE exercise group; a ratio equal to 1.0 indicates no difference in biomarker concentrations between the HIGH and MODERATE exercise groups

b. P for testing the HIGH-MODERATE group difference over 12 months from the linear mixed model, adjusted for biomarker level at baseline

c. Included *n* = 138 women for whom, across weeks 13–52 (at full prescription), average adherence in the exercise logs was 90–100% in the MODERATE group (135–150 min/week; *n* = 58) or ≥ 90% in the HIGH group (≥270 min/week; *n* = 80). Time at high intensity was defined as time exercising at an intensity of 60–80% heart rate reserve averaged for each participant over 52 weeks. Cut points for the stratified analysis were 60% of the prescribed durations, i.e., 90 min/week in the MODERATE group and 180 min/week in the HIGH group

d. Body mass index at baseline

e. Cut point of the baseline VO_2max_ level for the stratified analysis were the median value for all study participants (MODERATE and HIGH group)

f. Cut point of the baseline total body fat level for the stratified analysis were the median value for all study participants (MODERATE and HIGH group)

Stratifying results by baseline BMI, VO_2max_, and total body fat did not reveal any sub-group effects for VEGF (Table 2). Conversely, the result for FGF2 varied by baseline VO_2max_ and total body fat. Specifically, women with lower baseline physical fitness had an overall lower concentration of FGF2 in the HIGH group (TER 0.94, 95% CI 0.88, 1.00). However, this result was largely driven by the opposite direction of change at six months (8.2% increase in MODERATE group vs. 2.2% decrease in HIGH group). At 12 months, the HIGH group had a greater increase in FGF2 from baseline (8.2% vs 1.3% in MODERATE group). Likewise, the statistically significant TER for FGF2 observed among women with greater total body fat at baseline (TER 0.94; 95% CI 0.88, 1.00) was driven by the opposite direction of change at 6 months (5.8% increase in MODERATE group vs 2.2% decrease in HIGH group), while FGF2 showed a greater increase in HIGH group (8.5% vs 4.5% in MODERATE group).

## Discussion

Growth factors (i.e., FGF2 and VEGF) and their receptors (FGF2R and VEGFR), have been implicated in breast cancer tumorigenesis [10, 11], progression [4, 11], and metastasis [4]. However, the expression of specific FGF2 and VEGF polymorphisms has also been shown to be protective against developing breast cancer [12, 13]. In BETA, the greatest increases in FGF2 expression were observed with HIGH volumes of exercise in those with lower baseline physical fitness and higher total body fat whereas, VEGF expression was unaltered.

Our findings may be partially explained by the follow-up assessment timing and the somewhat differential nature of each growth factor’s pro-angio/arteriogenic action. In general, FGF2 and VEGF are important mediators of pro-angiogenic adaptations to repetitive exercise and act to increase tissue capillary density in response to hypoxic and inflammatory signaling [14]. However, it is well known that VEGF signaling is more central to supporting angiogenesis and neovascularization whereas FGF2 signaling plays a larger role in arteriogenesis and the maintenance of mature vessels [3, 15]. In general, chronically elevated growth factor levels may be considered pathologic – reflective of underlying tumor proliferative processes. However, in the context of exercise, transient growth factor increases can be viewed as normal physiologic hypoxia-driven adaptations – reflective of favorable vascular bed remodeling. While we would have expected to observe a transient increase in levels of these growth factors following the exercise intervention, in the context of the 12-month intervention within BETA, the six-month gap between follow-up assessments may have limited our ability to capture the transient spikes in the expression of these growth factors. Following an acute bout of exercise, the resulting increase in growth factor expression also appears to be transient – elevated after 7–10 days of training and returning to baseline after 28 days [16, 17].

In conclusion, high doses of supervised exercise did not impact levels of VEGF or FGF2. Additional mechanisms related to decreased breast cancer risk through altered adipose tissue require investigation.

## Abbreviations

BETA: Breast cancer and Exercise Trial in Alberta
BMI: Body mass index
FGF2: Fibroblast growth factor-2
ITT: Intention-to-treat
TER: Treatment effect ratio
VEGF: Vascular endothelial growth factor
